# Amplification mode differs along the length of the mouse cochlea as revealed by connexin 26 deletion from specific gap junctions

**DOI:** 10.1038/s41598-017-04279-3

**Published:** 2017-07-12

**Authors:** Victoria A. Lukashkina, Tetsuji Yamashita, Jian Zuo, Andrei N. Lukashkin, Ian J. Russell

**Affiliations:** 1 Sensory Neuroscience Research Group, School of Pharmacy and Biomolecular Sciences, University of Brighton, Brighton, BN2 4GJ UK; 2 Dept. of Developmental Neurobiology, St. Jude Children’s Research Hospital, Memphis, TN 38105 USA

## Abstract

The sharp frequency tuning and exquisite sensitivity of the mammalian cochlea is due to active forces delivered by outer hair cells (OHCs) to the cochlear partition. Force transmission is mediated and modulated by specialized cells, including Deiters’ cells (DCs) and pillar cells (PCs), coupled by gap-junctions composed of connexin 26 (Cx26) and Cx30. We created a mouse with conditional Cx26 knock-out (Cx26 cKO) in DCs and PCs that did not influence sensory transduction, receptor-current-driving-voltage, low-mid-frequency distortion-product-otoacoustic-emissions (DPOAEs), and passive basilar membrane (BM) responses. However, the Cx26 cKO desensitizes mid-high-frequency DPOAEs and active BM responses and sensitizes low-mid-frequency neural excitation. This functional segregation may indicate that the flexible, apical turn cochlear partition facilitates transfer of OHC displacements (*isotonic forces*) for cochlear amplification and neural excitation. DC and PC Cx26 expression is essential for cochlear amplification in the stiff basal turn, possibly through maintaining cochlear partition mechanical impedance, thereby ensuring effective transfer of OHC *isometric forces*.

## Introduction

**Figure 1:**
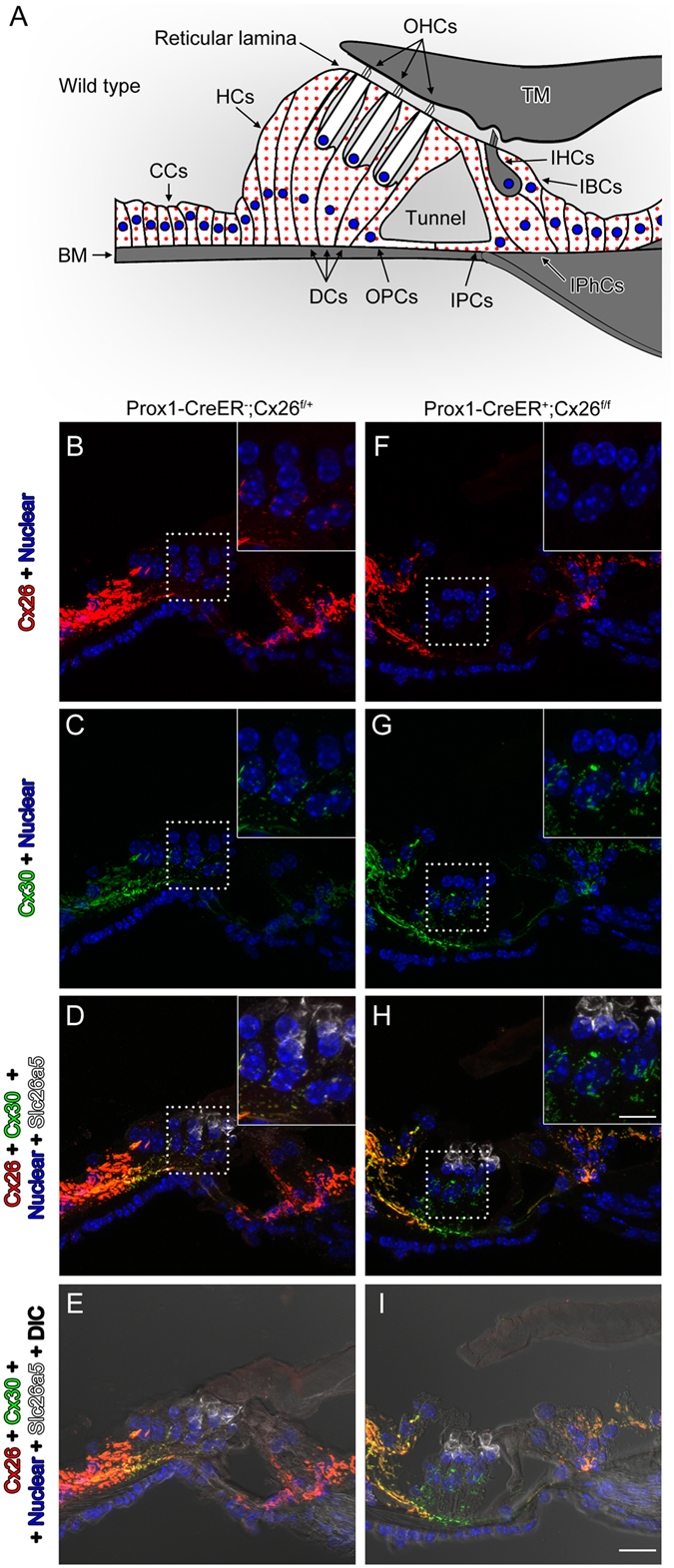
Specific deletion of Cx26 in Deiters’ and Pillar cells in *Prox1-CreER; Cx26* ^*f/f*^ cKO cochleae. **(A)** Diagram of cross section of the organ of Corti. The cells containing red dots indicate Cx26 expressing cells in wildtype cochleae. Slc26a5 (prestin)-positive OHCs (by white) and the remaining regions (in grey) are indicated. DCs are located underneath of OHCs. IPCs and OPCs are located between IHCs and OHCs. **(B**–**I)** Cx26 (in red), Cx30 (in green), and slc26a5 (in white) expressions in middle turns of either wildtype **(B**–**E)** or Cx26 cKO cochleae **(F–I)** at P32 are shown. Counter-staining of nuclei is indicated in blue. Dashed boxes indicate the regions of the insets. Scale bar expresses 5 µm (in **I**) and 2.5 µm (in an inset in **H**). [See Fig. 2 below].

Mechanical impedance matching in the mammalian cochlea^1, 2^ enables the transfer of voltage-dependent, prestin-driven forces^3^, between the OHCs and structures of the cochlear partition. OHCs interact with the DCs, outer pillar cells (OPCs) and the reticular laminar (Fig. 1A), which together provide a restraining, flexible, microtubule and actin-packed framework that enables OHCs to interact and exchange forces with the BM (Fig. 1A) to provide the exquisite sensitivity and frequency tuning of the mammalian cochlea^1, 4–6^. Apical-basal gradients of decreasing OHC length and increasing OHC axial stiffness are suggested to match the increasing apical-basal gradient of stiffness of the BM^1^. However, distribution of the OHC motor protein prestin^3^ remains constant throughout the length of the cochlea^7^. In contrast, prestin voltage sensor charge density has been found to increase with increasing frequency location despite constant prestin density^8, 9^. It remains to be discovered, however, if this gradient in charge density can account for the increase in gain of the cochlear amplifier from a few-fold at the apex of the cochlea to 1000-fold at the base^10^. The basal poles of OHCs are cupped in the DCs, which are coupled to each other and adjacent OPCs by large gap junctions^11^. The structural protein composition, including actin and tubulin, is far greater and more densely packed in basal turn PCs and DCs. These are structurally reinforced in bats with ultrasonic hearing and very stiff BM where, relative to the OHCs, the DCs and PCs are massive. Thus OHCs in the base of the cochlea appear to be preloaded through mechanical interaction with adjacent structures. This contrasts with the compliant apical turn BM of mole rats, that can detect infrasound, where the structure of PCs and DCs, where present, is gracile and with reduced structural protein expression^6^. The impression gained, therefore, from mechanical measurements and the functional organisation of the cochlea is that OHC voltage dependent forces in the apex of the cochlea are translated into displacements or *isotonic forces*, while those in the high-frequency base are translated into stiffness changes or *isometric forces* ^6^. This nonlinear frequency-dependent force, which boosts the sensitivity of cochlear responses to low-level sounds and compresses them at high levels, is known as the cochlear amplifier^12^.

The aim of experiments reported here, which has revealed difference in the mode of cochlear amplification between the apex and base of the cochlea, is to further understand the role in cochlear sensory processing of the large gap junctions that couple together the DCs and OPCs, but not the OHCs^11^. Each gap junction is formed by two interacting hemichannels (connexons) on neighbouring cells, each consisting of 6 connexin protein subunits, to permit the bidirectional flow of ions and signalling molecules. The hemichannels of type 1 fibrocytes of the spiral ligament, supporting cells of the sensory epithelium of the cochlea, the organ of Corti (OC), and cells within the basal cell region of the stria vascularis (SV) are formed of co-localised connexin 26 (Cx26 or GJB2) and Cx30 or GJB6^11^, deletions or mutations of which are responsible for most genetically-based hearing loss^13^. Measurements reported here, from mice with a conditional knockout (cKO) of Cx26 that deletes Cx26 from DC and OPC, reveals that conditional deletion of this protein has profoundly different effects in the basal and apical regions of the cochlea. Deletion seriously impairs cochlear amplification in the basal high frequency region of the cochlea, but may augment amplification in the apical region of the cochlea and signal transfer from the sensory-motor OHCs to the sensory inner hair cells (IHCs).

## Results

### Imunohistochemistry of Cx26cKO cochleae confirms targeted deletion of Cx26 from Deiters’ and outer pillar cells

**Figure 2:**
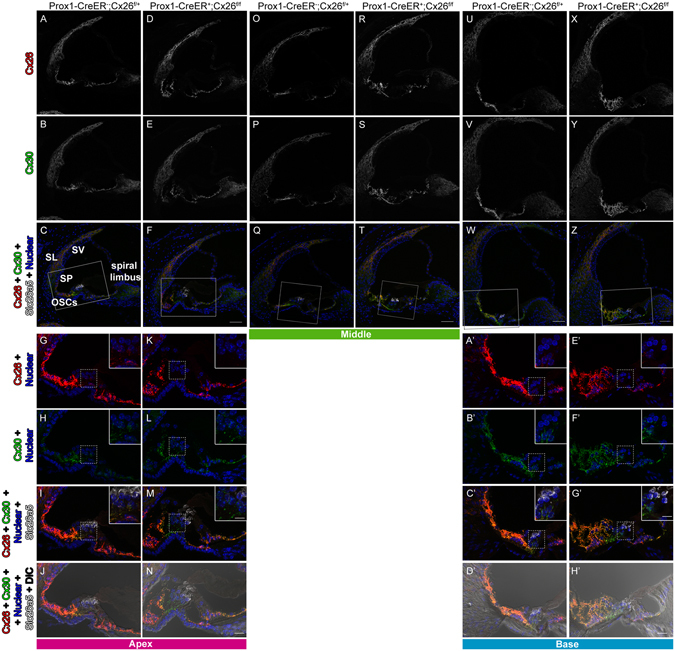
Specific deletion of Cx26 in Deiters’ and Pillar cells in *Prox1-CreER; Cx26* ^*f/f*^ cKO cochleae. (**A**–**H**’) Cx26 (in red), Cx30 (in green), and Slc26a5 (in white) expression in either wildtype (**A**–**C**, **G**–**Q**, **O**–**Q**, **U**–**W** and **A’**–**D’**) or Cx26 cKO cochleae (**D**–**F**, **K**–**N**, **R**–**T**, **X**–**Z**, and **E’**–**H’**) at P32 is shown. The representative confocal images were obtained from cochlear apical (**A**–**N**), middle (**O**–**T**), or basal (**U**–**H’**) turns. Counter-staining of nuclei is indicated in blue. Dashed boxes in **Q** and **T** indicate the areas shown in Fig. 1B–I. Enlarged pictures from dashed boxes in **C**,**F**,**W** and **Z** are shown in **I**,**M**,**C’**, and **G’**. The dashed boxes in **G**–**I**, **K**–**M**, **A’**–**C’** and **E’**–**G’** indicate the areas shown in the insets. Scale bar expresses 50 µm (in **F**,**T**,**W**, and **Z**), 5 µm (in **N** and **H’**), and 2.5 µm (in insets in **M** and **G’**).

To gain some understanding of the role of these gap-junctions in cochlear sensory processing, we created a mouse with conditional knockout (cKO) of Cx26. To delete the Cx26 gene in the DCs and pillar cells (PCs) *in vivo*, we generated *Prox1-CreER* ^+^ *; Cx26* ^*loxP/loxP*^ conditional KO (cKO) lines and injected tamoxifen daily once at P0 and P1. The specific Cre activities in DCs and PCs in *Prox1-CreER* ^*T2*+^ cochleae have been observed in many studies at different laboratories^14^. To confirm the Cx26 deletion, the protein expression was examined by immunostaining. Strong expression of Cx26 was observed in spiral ligament (SL), spiral prominence (SP), outer sulcus cells (OSCs), and spiral limbus in wild-type (WT) cochleae. The expression was also seen in basal cells in the stria vascularis (Fig. 2). In the OC, Cx26 expression was observed in the circumference of DCs, PCs, Hensens’ cells (HCs), Claudius cells (CCs), inner phalangeal cells (IPhCs), and inner border cells (IBCs) (Figs 1A–E and 2). These distributions were similar to those of Cx30 (Figs 1A–E and 2) and no obvious differences were observed from apical to basal turn of cochleae in distributions of either Cx26 or Cx30 (Figs 1A–E and 2). These observations were consistent with previous reports^15, 16^. In Cx26 cKO cochleae, Cx26 expression was abolished only in the circumference of DCs and PCs (Figs 1F–I and 2), consistent with specific Cre activities in DCs and PCs in *Prox1-CreER* ^+^ cochleae^14^ while Cx30 remained expressed (Figs 1F–I and 2). In contrast to other Cx26 mutations, which are not specific to the PCs and DCs and can influence development of the cochlea, resulting in a tunnel of Corti that remains closed^17^, the tunnel of Corti in the cochleae of Cx26cKO mice is morphologically indistinguishable from that in WT littermates (Figs 1E,I and 2N,H’).

### Targeted deletion of Cx26 from Deiters’ and outer pillar cells does not impair driving potential for OHC receptor currents or OHC sensory transduction

**Figure 3:**
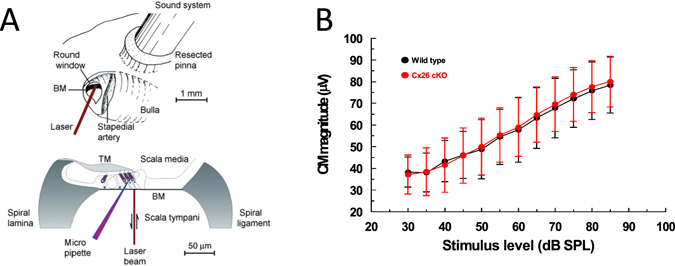
Round window CM are similar in WT and Cx26 cKO mice. (**A**) Techniques used to make electrophysiological and mechanical measurements from the cochlea (modified with permission^23, 24^). (**B**) CM magnitude as function of stimulus level for stimulation with 5 kHz tones (mean ± standard deviation, n = 5). Measurements from mice, 21–26 days post-partum.

The endocochlear potential (EP) of the scala media (Fig. 3A) is a major contributor to the driving voltage for the influx of receptor current^18^ through the mechanoelectrical transducer (MET) channels located at the tips of the stereocilia^19^. The EP was measured by advancing a sharp (50–70 MΩ) KCl filled micropipette through the round window (RW) membrane, BM, and OC into the scala media of the basal turn (Fig. 3A). The mean EP ± standard deviation for five mice was 111.0 ± 5.7 mV for WT and 110.4 ± 6.4 mV for homozygous Cx26 cKO mice and no significant difference was detected (unpaired t-test, two tail *p* value = 0.8795).

Cochlear amplification is initiated by the flow of current through mechanoelectical transducer (MET) channels located near the tips of the stereocilia which comprise the OHC hair bundles^19^. The driving voltage for this K^+^-dominated current is provided by the resting membrane potential (~−50 mV for OHCs)^20–22^ and the EP (~+ 110 mV, see above). The modulation of the MET current flow generated by the entire OHC population of the basal turn across the total electrical impedance of cochlear partition, as a consequence of acoustic stimulation, can be monitored by measuring the cochlear microphonic potential (CM). CM measured at the RW is dominated by basal turn OHC MET currents^23, 24^. CM was not used to assess cochlear amplification, sensitivity, or frequency selectivity, but to assess the MET of OHCs in the basal turn. The ear was therefore stimulated with 5 kHz tones, which is far below the 50 kHz–80+ kHz frequency range of the basal turn cochlear responses. This frequency was chosen because the entire basal turn of the cochlea should be displaced in unison^23^ and at saturating levels of the CM, all OHCs in the basal turn of the cochlea will contribute MET current to the CM^23, 24^. Stimulation with high frequency tones close to the sensitive frequency range of the basal turn^25^ will cause adjacent regions of the cochlear partition of the basal turn to move in opposite directions^23^, thereby causing complex phase augmentation and cancellation of the CM^23, 24^, which defeats the purpose of the measurement, which is simply to compare the functionality of MET in basal turn OHCs from Cx26 cKO mice and their WT littermates. CM will be absent or reduced if OHCs are lost or damaged. It is apparent from Fig. 3B, which includes measurements from mice used in the BM measurements described below, that CM level functions recorded from Cx26 cKO mice and their WT littermates are not significantly different (unpaired t test for each point in Fig. 3B, the two tailed *p* value < 0.9).

### High frequency hearing is desensitized and low-mid frequency hearing is sensitized in Cx26 cKO mice

**Figure 4:**
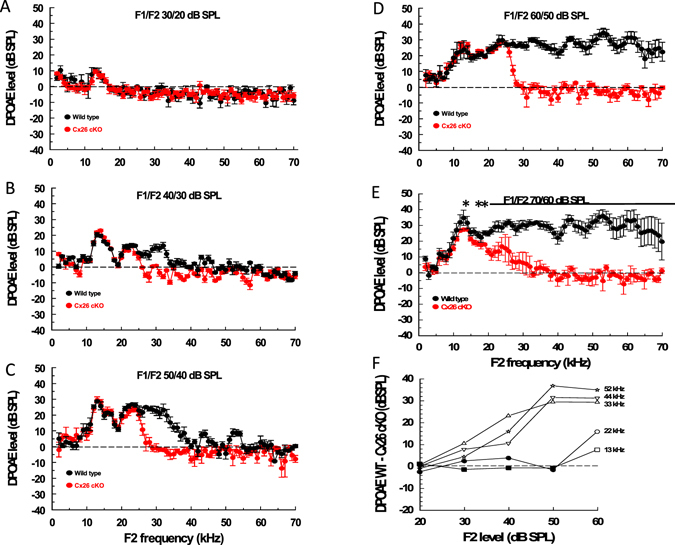
Low-frequency DPOAE responses are similar in WT and Cx26 cKO mice. (**A**–**E**) DPOAE isolevel audiograms (DPOAE 2f1−f2 magnitude, mean ± SD,) as a function of the f2 frequency (f2 levels: A = 20 dB SPL, B = 30 dB SPL, C = 40 dB SPL, D = 50 dB SPL, E = 60 dB SPL; f2/f1 ratio = 1.23; level of f2 set 10 dB below f1 level) from 5 WT (black symbols) and 5 Cx26 cKO (red symbols) mice. Dashed lines indicate measurement noise floor. Solid horizontal lines and asterisks indicate regions and points, respectively, where DPOAEs from WT and Cx26 cKO mice are significantly different (unpaired t-test, 0.05 two-tailed *p* value). F. Difference in mean magnitude between DPOAEs measured from WT and Cx26 cKO mice (obtained from data presented in Fig. 4A–E) at different frequencies, shown in Fig. 4F, as functions of f2 level. Solid symbols indicate measurements where there is no significant difference between measurements made from WT and Cx26 cKO mice (unpaired t-test, 0.05 two-tailed *p* value).

Distortion product otoacoustic emissions (DPOAEs) are nonlinear acoustical responses produced by the cochlea when stimulated simultaneously with two pure tones (f1 and f2, f1 < f2) with optimal frequency ratio and level differences^26^ (caption, Fig. 4). It is generally accepted that the recording of DPOAEs indicates that OHCs provide cochlear amplification. The background strain of Cx26 cKO mice is not particularly sensitive and DPOAE audiograms measured from both the WT and Cx26 cKO littermates with the level of f2 equal to 20 dB SPL are indistinguishable from each other. With f2 level set at 20 dB SPL, DPOAEs are at or close to the recording noise floor across the whole frequency range of the experiment (f2 = 2–70 kHz) except for a small peak close to 13 kHz (Fig. 4A). With increasing level of f2 from 20 dB SPL to 50 dB SPL, DPOAEs appear above the noise floor and the audiogram extends upwards in frequency with increasing SPL (Fig. 4B–D). With increasing f2 level the audiograms from WT mice extend upwards in frequency to the upper limit of the f2 stimulus range (70 kHz). The audiograms of Cx26 cKO mice are indistinguishable from those of their WT littermates for frequencies just below 25 kHz at all f2 levels at or below 50 dB SPL. Above this frequency, DPOAEs recorded from Cx26 cKO mice decline rapidly to the noise floor at ~30 kHz (Fig. 4B–D). As can be seen, this rapid decline in DPOAEs is a step-wise and not a gradual, frequency-dependent process. Lines and asterisks in Fig. 4 indicate frequency regions where the audiograms of Cx26 cKO mice and their WT littermates are significantly different (unpaired t-test, 0.05 two-tailed *p* value). The audiograms of both the WT and Cx26 cKO littermates change at f2 levels of 60 dB SPL. The audiograms of the WT mice show greater variation at the highest frequencies, as indicated by the increased size of the standard deviations (Fig. 4E). Audiograms from the Cx26 cKO littermates do not closely resemble those of their WT littermates, but decline gradually, and with variability between preparations (note standard deviations, Fig. 4E) from 13 kHz to the noise floor at 30 kHz. Similarities and differences in the audiograms recorded from WT mice and the Cx26 cKO littermates are shown in Fig. 4F. This represents the difference (WT–Cx26 cKO) of the mean amplitude values of the audiograms at 5 representative f2 frequencies at all f2 levels between 20 and 60 dB SPL. For levels at 50 dB SPL and below, there is no significant difference in DPOAEs measured at frequencies of 13 kHz and 22 kHz. For frequencies 33 kHz, 44 kHz and 52 kHz, DPOAEs recorded from WT mice are larger than those of their Cx26 cKO littermates, for which DPOAE is at the noise floor, for all f2 levels above 20 dB SPL.

**Figure 5:**
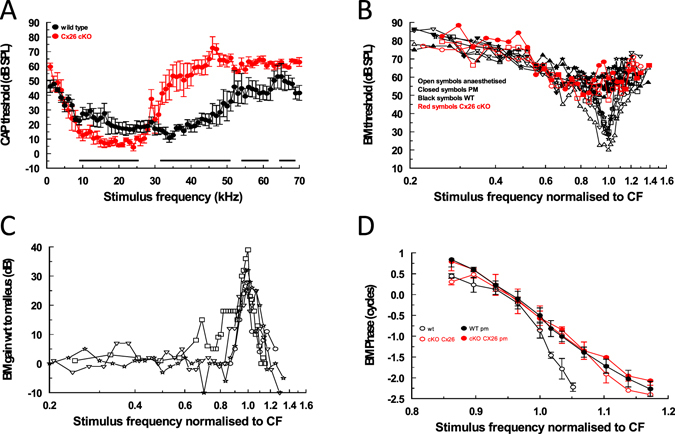
High-frequency hearing is desensitized and detuned and low-mid frequency hearing is sensitized in Cx26 cKO mice. (**A**) Compound action potential (CAP) audiogram (mean ± SD of CAP detection threshold as a function of stimulus frequency) from 5 WT (black symbols) and 5 Cx26 cKO (red symbols) mice. Solid horizontal lines indicate regions where CAP thresholds of WT and Cx26 cKO mice are significantly different (unpaired t-test, 0.05 two-tailed *p* value). (**B**) Threshold (0.2 nm) frequency tuning curves measured from the 54 kHz–59 kHz frequency region of the basal turn BM from 5WT, and 2 Cx26 cKO mice, anaesthetized and post-mortem. The frequency axes represent the stimulus frequency normalised to the characteristic frequency (CF) of the measurement location. Different symbols indicate different preparations. (**C**) Gain of BM displacement as a function of stimulus frequency (normalised to the CF of the measurement location) relative to malleus displacement for 4 WT mice. (**D**) BM phase as a function of stimulus frequency (normalised to the CF of the measurement location) measured from the 54 kHz–59 kHz frequency region of the basal turn BM from 5WT, and 5 Cx26 cKO mice, anaesthetized and post-mortem. All mice used in the study were 21–26 day post-partum.

The DPOAE measurements are supported in part by RW measurements of the compound action potential (CAP, Fig. 5A). The CAP is due to the synchronized activity of the auditory nerve fibres to tones that, at CAP threshold, are close in frequency to the characteristic frequency of the IHCs, which form synapses with the fibres^27^. For frequencies between 10–27 kHz, the CAP threshold is significantly lower in Cx26 cKO mice than in their WT littermates (unpaired t test for each point between 9–27 kHz, Fig. 5A, two-tailed *p* value < 0.05). For frequencies >30 kHz, CAP thresholds of WT mice are significantly lower than the thresholds of Cx26 cKO littermates. Frequency regions of significant difference between the CAP frequency threshold curves of Cx26 cKO mice and their WT littermates are shown by lines in Fig. 5A. Larger standard deviations of measurements on the high-frequency slopes of the CAP audiograms of both the WT and Cx26 cKO mice are observed because of variation in the upper range of high-frequency sensitivity in both mouse types.

### Basilar membrane responses in the basal turn of the cochlea of CX26 cKO mice are insensitive and broadly tuned

Measurements of BM displacement, gain and phase were made from the basal turns of the cochleae of WT and homozygous Cx26 cKO littermates using a self-mixing, laser diode, interferometer, the beam of which was directed through the unopened RW membrane (Fig. 3A). Threshold tuning curves and maximum gain of BM displacement with respect to measurements made post-mortem from WT mice are similar to those obtained from WT mice on previous occasions^5, 28, 29^. From measurements made at BM characteristic frequency (CF) locations between 54 kHz and 59 kHz in 5 mice, the mean ± standard deviation of the threshold was 26.6 ± 3.5 dB SPL, the sharpness of the tuning curve (Q_10 dB_ = CF/ bandwidth 10 dB from the tip) was 8.7 ± 1.6 (Fig. 5B), and the maximum gain measured from the WT mice was 32.5 ± 5.2 dB (Fig. 5C). Examples of threshold tuning curves from homozygous Cx26 cKO mice are shown in Fig. 5B. The tuning curves (red open symbols) are insensitive, and appear not to differ from measurements made post mortem from WT and Cx26 cKO mice (solid symbols).

BM phase measurements were made from the 54–59 kHz BM location in response to tones between 50 kHz and 70 kHz (Fig. 5D). Tone levels were set at 40 dB SPL for WT mice. At this level it is anticipated that active cochlea amplification should contribute to BM vibration^10^. The relationship between BM displacement and stimulus level (dB SPL) is linear for the Cx26 cKO and post-mortem mice, and so the stimulus levels were increased to 80 dB SPL to order to obtain reliable phase responses over the entire frequency range. Phase roll-off is steeper (slopes of 232 ± 14 degrees/kHz, n = 5) for frequencies just below and above the CF (1.0 on the normalized frequency axis) for WT mice (Fig. 5D, black open symbols) than it is (slopes of 85 ± 7 degrees/kHz, n = 5) for the Cx26 cKO mice (Fig. 5D, red open symbols). This phase difference disappears post mortem, when the BM phase frequency-dependence of both WT (Fig. 5D, black closed symbols) and Cx26 cKO mice (Fig. 5D, red closed symbols) resemble that of the living Cx26 cKO mice.

## Discussion

Based on measurements of DPOAE audiograms, which provide a direct indicator of cochlear sensitivity at the level of the OHCs, there is no significant difference in cochlear sensitivity between Cx26 cKO mice and their wild type littermates for f2 frequencies between 2 kHz, which is close to the low frequency range of cochlear mediated hearing in mice^30, 31^, and ~25 kHz at stimulus (f2) levels of 50 dB SPL and below. For frequencies between ~25 kHz and 30 kHz, DPOAE level of Cx26 cKO mice declines, almost step-wise, and disappears into the recording noise floor. Over the frequency range of 10–27 kHz, the threshold of excitation of afferent fibres, as indicated by the threshold of CAP audiograms, is sensitized in Cx26 cKO mice, possibly through increased excitation of the IHCs. Thus, loss of expression of Cx26 in DCs and PCs has no consequences for excitation and amplification of OHCs for frequencies below 25 kHz in the Cx26 cKO mouse cochlea, but it has profound consequences for cochlear amplification above this frequency place. According to our measurements, therefore, specific deletion of Cx26 in DCs and PCs desensitizes high-frequency hearing and sensitizes low-frequency hearing in Cx26 cKO mice. The frequency-dependent decline from ~13 kHz of DPOAEs recorded from Cx26 cKO mice for f2 levels of 60 dB SPL indicates a vulnerability of cochlear sensitivity to moderately loud sounds when Cx26 is not expressed in PCs and DCs. The behaviour is reminiscent of intracellular OHC responses to and following exposure to brief loud sounds^32^. The DPOAEs of Cx26 cKO mice are not sustained in magnitude and do not follow those of DPOAEs recorded from WT littermates, but decline slowly for frequencies between ~13 kHz–30 kHz. In this respect, it has been reported that reduced Cx26 expression in the mature cochlea increases susceptibility to noise-induced hearing loss in mice^33^. These phenotypes appear without any other apparent changes in Cx26 and Cx30 expression from that seen in WT littermates. This data is confirmed indirectly by the discovery that loss of the Cx26 expression in DCs and PCs appears to have little effect on the overall electrical properties of the cochlea, including the OHC MET currents in the basal turn of the cochlea and on the electrical impedance of the MET current return pathways^22^. Accordingly, loss of Cx26 expression in DCs and PCs influences neither the driving voltage for the receptor currents (i.e. the EP) nor mechanoelectrical transduction of OHCs in the basal turn of the mouse cochlea as evidenced by the CM, which is similar in characteristics and magnitude to that reported previously^10, 23, 24, 28, 29, 34^. It would appear, therefore, from our DPOAE and CAP measurements that the loss of Cx26 in gap-junctions between the DCs and PCs is associated with loss of cochlear amplification, but only for frequencies above ~25 kHz.

Our data differ from that published previously on the same Cx26 cKO mouse strain, over a limited frequency range^25^. Zhu *et al*.^25^ found that DPOAEs recorded for an f_0_ of 20 kHz (f2 = 21.91 kHz) from Cx26 cKO mice were, by comparison with WT littermates, reduced by 15.3 ± 6.92, 30.2 ± 4.47 and 30.9 ± 7.89 dB for stimulus levels of 40, 50 and 60 dB SPL. By comparison, we found no significant difference between DPOAEs recorded from Cx26 cKO and WT littermates for f2 = 22 kHz at f2 levels of 40 and 50 dB SPL and a reduction of 11.33 ± 1.86 dB SPL when the f2 level was 60 dB SPL. When f2 was set at 16 kHz and 60 dB SPL, we measured a reduction in DPOAE (WT - Cx26 cKO) of 6.98 ± 1.95 dB SPL. Zhu *et al*. recorded a reduction of 15.8 ± 7.23 for f_0_ of 16 kHz and 60 dB SPL. We suggest, above, that the difference in sensitivity of DPOAEs recorded in Cx26 cKO mice is due to a vulnerability of cochlear sensitivity to moderately loud sounds when Cx26 is not expressed in PCs and DCs. It could be that the mice used in Zhu *et al*.’s measurements are especially vulnerable to sounds at moderate levels. An indication that this might be the case are differences in EP recorded from mice in our experiments (111.0 ± 5.7 mV for WT and 110.4 ± 6.4 mV for homozygous Cx26 cKO mice) and EPs, almost 20 mV lower, recorded in Zhu *et al*.’s experiments (92.0 ± 2.31, WT and 91.5 ± 0.50 mV homozygous Cx26 cKO). Cochlea with low EP are particularly sensitive to noise-induced hearing loss^35^. This explanation may also account for differences in the neural responses (CAP threshold recorded in our experiments and ABR measurements in Zhu *et al*.’s experiments). CAP thresholds are on average 20.12 dB SPL more sensitive for the 5 frequencies (8, 16, 24, 32, and 40 kHz) at which Zhu *et al*. measured ABR thresholds. This is an expected consequence of low EP^35^. Under these circumstances our observation of an enhanced sensitivity of neural thresholds at 8, 16, and 24 kHz of Cx26 cKO mice over those from WT mice would likely be missed in Zhu *et al*.’s experiments.

BM responses in the basal turn of the cochlea of Cx26 cKO mice are insensitive and broadly tuned and are unchanged post-mortem, closely resembling the post-mortem BM responses of WT mice. It is apparent, therefore, that Cx26 expressed exclusively in DCs and PCs contributes towards the mechanical properties of the active, not the passive cochlea. The presence of Cx26 in the large gap-junctions directly, or indirectly, enables OHCs to generate the feedback essential for providing the large cochlear gain required for sensory processing in the basal, high frequency, turn of the cochlea. This finding contrasts with that produced by a point mutation to Cx30, which together with Cx26, forms gap-junctions. This Cx30 mutation produces changes in the passive mechanical properties of the cochlea while preserving the active component^22^.

Could the apical-basal, cochlea-position-dependent effects on cochlear sensitivity caused by the Cx26 cKO be due to a position-dependent expression of Cx26? In recent studies^18, 36, 37^ we showed Cre activities in Prox1CreER cochleae in multiple reporter lines (tdTomato and EGFP) that most likely represent the true patterns of Cre-mediated deletion of multiple genes. With respect to the data reported here, 60% of inner PCs were Cre+ at the apex, 27% at the middle, and 13% at the base of the cochlea. By comparison, 80–90% of OPCs and DCs of rows 1–3 (1 being the closest to the IHCs) were Cre+ at the apex and middle and only 60–70% at the base. It is very likely, therefore, that Cx26 is ablated with mosaic patterns in PCs and DCs and Cx26 is deleted in more PCs and DCs in the apical turns than in the basal turn of cochleae. Even so, despite the smaller decrease in Cx26 in the basal turn compared to that in the apical turn of the Cx26 cKO mouse, cochlear amplification appears to be significantly more dependent on the level of Cx26 in PCs and DCs in the base of the cochlea than in the apex, where it is apparently unaffected by a much larger Cx26 deletion. Because the deletions in Cx26 were confined to PCs/DCs and no other features were induced, these phenotypes observed must primarily be due to changes in Cx26-containing gap-junctions in DCs and PCs that normally play critical roles in the cochlea. This primary effect of the mutation could trigger secondary effects that are strongly implicated in, or perhaps dominate, the apical-basal differences in observed cochlear sensitivity of Cx26 cKO mice.

The basis for the desensitization and, indeed, low-frequency sensitization is unknown. It is tentatively suggested that changes in the properties of the gap-junctions, including their conductance, leads to changes in the dynamic mechanical properties of the supporting cells, and these changes are the primary cause of both the mid-high-frequency desensitization and low-frequency sensitization of cochlear responses. Changes in gap-junction conductance could lead to changes in the flow of ions and intercellular signalling molecules between supporting cells, and hence in their local intracellular levels^38^. With respect to this, it has been shown that conductance of the large gap-junctions is mechanically sensitive^39^ and that isolated DCs exhibit calcium- dependent motility^40, 41^. These two observations form a basis for a potential feedback mechanism in DCs. A change in the kinetics of intercellular calcium flow between supporting cells could influence DC motility. The motility could further affect intracellular calcium flow via changes in conductance of mechanically sensitive gap-junctions. Thus, any change in gap-junction conductance as a consequence of the Cx26 cKO could further alter intracellular ion composition as a consequence of changes in DC motility due to mechanical sensitivity of gap-junctions.

It is unlikely that the Cx26 cKO interferes with the important role of potassium recycling^42, 43^. Passive CM was unaffected by the Cx26 cKO. A consequence of reduced potassium recycling should be increased potassium concentration in the fluid-filled spaces of the organ of Corti^44^ accompanied by a decline in OHC receptor potential^45^ and a similar decline in CM^46^, which was not observed.

How can changes in dynamic mechanical properties of DCs, and perhaps PCs, influence cochlear amplification in Cx26 cKO mice? It has frequently been proposed^47^ that an essential role for the DCs and PCs is to provide a mechanical framework that mediates the exchange of forces between the OHCs and the cochlear partition. A proposed role for the DCs and PCs is to ensure adaptive mechanical impedance matching to the rest of the cochlear partition^2, 48^. This task may be critical in the stiff basal turn of the cochlea. Not only must the DCs and PCs be able to transmit sufficient energy from the motile OHCs into the region of the cochlear partition where the BM is stiffest, but also the mechanical impedance of DCs and PCs must be optimized to assure the precise timing of this transmission. Timing is essential for accomplishing the 1000-fold gain of the cochlear amplifier in the basal turn of the cochlea^10^.

Why should proposed changes in the dynamic mechanical properties of supporting cells have no apparent influence on amplification in the apical turns of the cochleae Cx26 cKO mice? Perhaps OHC motility delivers energy to apical and basal turn cochlear partitions in different ways? To optimise mechanical impedance matching^2, 48^ in the stiff basal turn of the cochlea, the framework provided by DCs and PCs must be rigid so that forces delivered by the OHCs are almost isometric^6^. The cochlear partition moves more in the apical region. DCs and PCs are more gracile, may enable the transfer of isotonic rather than isometric forces, and may even be absent in low-frequency hearing specialists^2, 6^. Differences between cochlea apex and base in the ways OHCs are mechanically coupled to the cochlear partition may account for why the high-frequency region of the cochlea is so susceptible to cochlear insults including noise and hypoxia.

Specific deletion of Cx26 from PCs and DCs may have secondary consequences for OHC voltage dependent motility. From ultrastructural and *in vitro* studies it appears that the DCs and PCs act together as cytoskeletal cages^49, 50^ that can control the OHC turgor pressure and hence the operating point for prestin-mediated, voltage-dependent, force generation^39, 51^ which presumably must be optimal for providing the feedback essential for cochlear amplification^10^. One suggestion is that the control of OHC turgor pressure is disrupted by the Cx26 cKO^25^. Surprisingly, to us, the peak of nonlinear-capacitance is shifted negatively in OHCs isolated from cochleae with Cx26 cKO^21^. It is presumed that the harvested OHCs^21^ must come from cochlear apical-middle turns because OHCs from the basal turn do not usually survive isolation^21^. It would seems that nonlinear-capacitance shift, if it occurs in Cx26 cKO mice used in our measurements, has no consequences for amplifying low-mid frequencies because we see no change in apical-middle-turn cochlear sensitivity. It remains to be seen if desensitization of high-frequency, basal-turn cochlear responses in Cx26 cKO mice is due to a peak shift in OHC nonlinear-capacitance.

Another secondary effect of the Cx26-cKO could be to change the activity and distribution of the motor protein prestin, which has been reported to be activity dependent^52^. However, prestin expression and maximum charge, which reflects the functional expression of prestin at the OHC lateral wall, is unchanged by the Cx26 cKO^25^. However, such changes can only be directly measured in the apical turns in adult mice where sensitization occurs in Cx26 cKO mice reported here. Currently, it has not been possible to measure such changes in the basal turn of the cochlea, which is strongly desensitized in Cx26 cKO mice.

To conclude, Cx26 cKO from gap-junctions between PCs and DCs has opposite effects on the sensitivity of the cochlea at its base and apex. The active, but not the passive, mechanics of the cochlear partition in the mid-high frequency region of the cochlea is strongly compromised. We suggest that in this region Cx26 contributes to the active mechanical properties of the cochlea that are important for enabling amplification. In the mid-low frequency region of the cochlea the Cx26cKO has no apparent influence on OHC responses, as indicated by lack of observable difference in DPOAEs measured from Cx26cKO mice and their WT littermates. However, the sensitivity of CAP thresholds is significantly increased, thereby indicating that the active mechanics of the cochlear partition is changed by the Cx26cKO to enhance IHC excitation.

## Materials and Method

### Cx26 cKO mouse generation and genotyping

The Animal Care and Use Committees of St. Jude Children’s Research Hospital approved the immunostaining and morphological analysis protocols performed in this study. Mice were housed under a 12 h light/dark cycle with free access to food and water. *Prox1-CreER* ^*T2*+^; *Cx26* ^*loxP/loxP*^ cKO lines were generated by crossing *Cx26* ^*loxP/*+^ mice with *Prox1-CreER* ^*T2*+^; *Cx26* ^*loxP/*+^ mice. The genotyping for the Cx26 floxed allele and *Prox1-CreER* ^*T2*^ transgene was performed as previously described^53, 54^. Induction of Cre activities was performed as previously described^14^. All procedures involving animals performed at the University of Brighton were in accordance with UK Home Office regulations with approval from the local ethics committee.

### Immunofluorescent staining and confocal microscopy

Immunofluorescent staining was performed using cochlear frozen sections prepared from mice at P32. Briefly, inner ears were fixed with 4% paraformaldehyde in 0.1 M phosphate buffer (pH 7.4) for 4 hours at room temperature, decalcified in 10% EDTA at 4 °C overnight, and embedded in O.C.T compound (Tissue-Tek, Sakura Finetek USA, Inc., Torrance, CA, USA). The frozen inner ears were sectioned at 12 µm and incubated with monoclonal mouse anti-Cx26 (Thermo Fisher Scientific, San Jose, CA, USA, Cat# 33-5800), polyclonal rabbit anti-Cx30 (Thermo Fisher Scientific, Cat# 71-2200), and polyclonal goat anti-prestin (Santa Cruz Biotechnology, Santa Cruz, CA, USA, Cat# sc-22692). The immunoreactivity was visualized using secondary Alexa Fluor® 488, 568, or 647 conjugated antibodies (Thermo Fisher Scientific) and counter-staining of nuclei was performed using 4′,6-diamidino-2-phenylindole (DAPI, SIGMA, St-Louis, MO, USA). The prepared samples were analyzed using a LSM710 confocal laser scanning image system (Carl Zeiss, Jena, Germany).

### Physiological Recordings

Mice 21–26 days post-partum, were anesthetized with ketamine (0.12 mg/g body weight i.p.) and xylazine (0.01 mg/g body weight i.p.) for nonsurgical procedures or with urethane (ethyl carbamate; 2 mg/g body weight i.p.) for surgical procedures. The animals were tracheotomized, and their core temperature was maintained at 38 °C. To measure BM displacements, CM and CAPs (Fig. 3A), a caudal opening was made in the ventro-lateral aspect of the right bulla to reveal the RW. CM and CAPs were measured from the RW membrane by using glass pipettes filled with artificial perilymph, with tip diameters of 50 to 100 μm (recording bandwidth >30 kHz). EP was measured using sharp micropipettes (70–100 MΩ, 3 M KCl, filled) pulled from 1 mm O.D., 0.7 mm I.D quartz glass tubing on a Sutter P-2000 micropipette puller (Sutter Instrument Novato, CA 94949, USA). Electrodes were advanced using a piezo activated micropositioner (Marzhause GMBH). The pipette tip was inserted through the RW membrane and into the BM, close to the feet of the OPCs, under visual control. The electrode was stepped slowly through the OC. The first cells to be encountered had resting potentials ≤−80 mV, could be held for several minutes and were assumed to be supporting cells. Other cells encountered immediately before penetrating the scala media had resting potentials of ~−50 mV and could be held for seconds to several minutes. These were presumed OHCs. Loss in sensitivity of the preparation was determined by changes in CM threshold. Losses were never encountered as a consequence of intracellular penetration with the electrode. Experiments were terminated immediately there was any loss in CM threshold (≥5 dB SPL) due usually to change in the condition of the preparation. Signals were amplified with laboratory designed and built amplifiers with a recording bandwidth of DC–100 kHz. Sound was delivered via a probe with its tip within 1 mm of the tympanic membrane and coupled to a closed acoustic system comprising two MicroTechGefell GmbH 1-inch MK102 microphones for delivering tones and a Bruel and Kjaer (www.Bksv.co.uk) 3135 0.25-inch microphone for monitoring sound pressure at the tympanum. The sound system was calibrated *in situ* ^55^ for frequencies between 1 and 70 kHz by using a laboratory designed and constructed measuring amplifier, and known sound pressure levels (SPLs) were expressed in dB SPL with reference to 2 × 10^−5^ Pa. White noise and tone pulses with rise/fall times of 0.2 ms were synthesized by a Data Translation 3010 (Data Translation, Marlboro, MA) data acquisition board, attenuated, and used for sound-system calibration and the measurement of electrical and acoustical cochlear responses. To measure DPOAEs, primary tones were set to generate 2f1−f2 distortion products at frequencies between 1 and 50 kHz. DPOAEs were measured for levels of f1 ranging from 10 to 80 dB SPL, with the levels of the f2 tone set 10 dB below that of the f1 tone. System distortion during DPOAE measurements was 80 dB below the primary tone levels. Tone-evoked BM displacements were measured by focusing the beam of a self-mixing, laser-diode interferometer through the RW membrane to form a 20-μm spot on the centre of the BM in the 48- to 61-kHz region of the cochlea. The interferometer was calibrated at each measurement location by vibrating the piezo stack on which it was mounted over a known range of displacements^56^. Tone pulses with rise/fall times of 1 ms were used during CAP and BM measurements. Stimulus delivery to the sound system and interferometer for calibration and processing of signals from the microphone amplifiers, microelectrode recording amplifiers, and interferometer were controlled by a DT3010/32 (Data Translation, Marlboro, MA) board by a PC running Matlab (The MathWorks, Natick, MA) at a sampling rate of 250 kHz. The output signal of the interferometer was processed using a digital phase-locking algorithm, and instantaneous amplitude and phase of the wave were recorded.

All measurements were performed blind. Randomization was not appropriate because we had no foreknowledge of the genotype, although we could guess it from the phenotype. Genotypes (WT and homozygous Cx26 cKO) were determined beforehand for age-matched animals, and the physiologists were given the animals and kept blinded to the genotypes. DPOAEs were measured from mice under ketamine xylazine anaesthesia. From these measurements, it was easy to predict which ones were from WT and which were Cx26 cKO mice. These mice were reserved for further CAP, CM, and BM measurements under terminal anaesthesia. Measurements were made from each animal in a litter and data was analysed at the end of each set of measurements. When all measurements had been made from a particular litter, the tissue was genotyped again. Through using littermates and standardizing the phenotype of the background strain, we reduce variability in our data due to age differences and variation in background strain. This permitted us to greatly reduce the numbers of mice we use in our experiments to get statistically significant results. Thus only sufficient numbers of measurements were made to obtain statistically significant differences. Experiments were terminated (<5% of all measurements) if the physiological state of the preparation changed during a measurement and data from the measurement was excluded. All relevant data are available from the authors.
